# Time to anti-retroviral therapy among people living with HIV enrolled into care in Myanmar: how prepared are we for ‘test and treat’?

**DOI:** 10.1080/16549716.2018.1520473

**Published:** 2018-11-30

**Authors:** Kyaw Zin Linn, Hemant Deepak Shewade, Kyaw Ko Ko Htet, Thae Maung Maung, San Hone, Htun Nyunt Oo

**Affiliations:** a National AIDS Programme, Department of Public Health, Ministry of Health and Sports, Nay Pyi Taw, Myanmar; b International Union Against Tuberculosis and Lung Disease (The Union), South-East Asia Office, New Delhi, India; c International Union Against Tuberculosis and Lung Disease (The Union), Paris, France; d Department of Medical Research, Pyin Oo Lwin branch, Pyin Oo Lwin, Myanmar; e Department of Medical Research, Yangon Head Quarter, Myanmar

**Keywords:** ART initiation, time to ART, attrition, HIV care cascade, SORT IT, operational research

## Abstract

**Background**: Among people living with HIV (PLHIV) enrolled into care, time to anti-retroviral therapy (ART) has not been studied in Myanmar. To inform progress, we conducted this operational research among treatment-naive PLHIV (≥18 years) enrolled during a period of three years (2014–2016) at Pyin Oo Lwin, Myanmar.

**Objectives**: To determine (i) the time from HIV diagnosis to ART initiation (time to ART) and associated factors and (ii) the association between time to ART and attrition (loss to follow-up and death) from ART care.

**Methods**: This was a retrospective cohort study involving a record review of secondary programme data. The PLHIV were followed up to 5 December 2017 for ART initiation and up to 31 March 2018 (date of censoring) for attrition during ART.

**Results**: Of 543 enrolled, 373 (69%) were found to be eligible and initiated on ART. Of 373, 245 (67%) were initiated within 6 weeks of enrolment. The median enrolment delay (from diagnosis) was 4 (IQR: 1, 14) days and median ART initiation delay (from ART eligibility) was 20 (IQR: 13, 36) days. The median time to ART (excluding the time interval in pre-ART care) was 29 (IQR: 18, 55) days and was significantly long in those with prevalent TB and CD4 count ≥ 500/mm^3^ at enrolment. Among 373, the annual incidence density of attrition was 12.8% (0.95 CI: 10.2, 15.7). Attrition was common in first 100 days. Time to ART (after excluding time interval in pre-ART care) was not significantly associated with attrition.

**Conclusion**: The programme appears to be on track to initiate ART as soon as possible in a ‘test and treat’ scenario (implemented since September 2017) subject to interventions to reduce ART initiation delay.

## Background

Globally, the new human immunodeficiency virus (HIV) infections and acquired immunodeficiency syndrome (AIDS)-related deaths are declining. However, in 2016 there were 1.8 million people newly infected with HIV and 1 million AIDS-related deaths. Of 36.7 million people living with HIV (PLHIV), 19.5 million are on anti-retroviral therapy (ART) [1]. With the aim of ending the HIV epidemic by 2030, the Joint United Nations Programme on HIV/AIDS in 2013 set an ambitious 90-90-90 target: (i) 90% of PLHIV know their status, (ii) 90% of PLHIV who know their status receive ART, (iii) 90% of PLHIV on ART have suppressed viral loads by 2020 [2,3].

**Figure 1.**
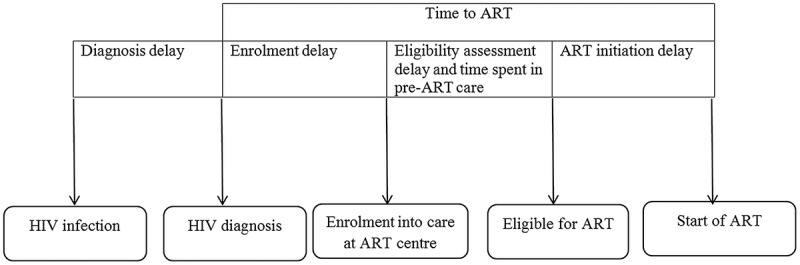
Conceptual framework of delays in HIV care cascade, Myanmar, 2014–2016*. HIV = human immunodeficiency virus, ART = anti-retroviral therapy *Eligibility assessment delay and time spent in pre-ART care will not be applicable in a ‘test and treat’ setting (implemented since September 2017).

The HIV care cascade before ‘test and treat’ included four stages: (i) from HIV diagnosis to enrolment; (ii) enrolment to ART eligibility (pre-ART care); (iii) eligibility to ART initiation; (iv) retention while on ART [4]. The corresponding possible delays are: HIV diagnosis delay, enrolment delay, delays during eligibility assessment and pre-ART care, and ART initiation delay (Figure 1). Time to ART (from HIV diagnosis) has been recommended as an indicator to supplement existing HIV cascade indicators [4,5].

Studies globally have reported a long time to enter into care and/or initiate ART after HIV diagnosis [5–11]. The enrolment and ART initiation delay (Figure 1) can be due to underlying factors like client readiness/refusal, physical disability and physician concerns to defer ART initiation during the treatment of co-morbidities especially anti-TB treatment [6,8,10].

Myanmar is one of the 35 countries that account for 90% of new HIV infections globally and one of the 6 countries in Asia identified as a priority for the global fast-track strategy to end the epidemic by 2030. The HIV epidemic is concentrated among key populations and the prevalence in the adult population is 0.6% (2016) [12]. Between 2014 and 2016, Myanmar has done a commendable job in increasing the access to ART (from 85,600 to 130,000), which is one of the factors that has resulted in reductions in annual new infections (12,000 to 11,000) and AIDS-related deaths (9700 to 7800) [12,13].

Studies from Myanmar have documented low retention in pre-ART care and high retention once on ART [14–16]. PLHIV initiated on ART had low CD4 counts at baseline indicating delays before ART initiation [14–16]. Hence, there was a need for systematic assessment of time to ART and associated factors which has not been studied in the National AIDS Programme (NAP) setting. Globally, the association of the time to ART with attrition has not been studied.

With the World Health Organization (WHO)’s ‘test and treat’ strategy, which Myanmar currently implements (since September 2017), the delay during eligibility assessment and pre-ART care (enrolment to ART eligibility) becomes irrelevant as all enrolled PLHIV are eligible for ART [17,18]. A new HIV care cascade has therefore been recommended where the stage from enrolment to eligibility has been removed and an additional stage of early retention in ART care introduced [19]. Therefore, time to ART will now include enrolment delay and ART initiation delay. As Myanmar gears up to implement the ‘test and treat’ strategy, reduction in enrolment and ART initiation delays is key to ensure a short time to ART after HIV diagnosis [20].

Hence, to inform progress we conducted this operational research during a period of three years (2014–2016) before implementation of ‘test and treat’. We aimed to determine (i) the time from HIV diagnosis to ART initiation (time to ART) and associated factors, and (ii) the association between time to ART and attrition (loss to follow-up and death) from ART care in Pyin Oo Lwin, Myanmar.

## Methods

### Study design and participants

This was a retrospective cohort study involving a record review of secondary programme data. We included all PLHIV enrolled into care in a Pyin Oo Lwin ART centre (under NAP) between 1 January 2014 and 31 December 2016. PLHIV < 18 years of age and pregnant/post-partum women (at enrolment) were excluded. PLHIV re-enrolled into care after a previous episode of loss to follow-up (either during pre-ART care or during ART care) and PLHIV transferred in (either during pre-ART or ART care) from other ART centres were also excluded.

### Setting

#### General setting

Myanmar is situated in south-east Asia and has an estimated population of 51 million, of whom 70% live in rural areas. It has 1 union territory and 14 states and regions. They are administratively divided into districts and further into 412 townships/sub-townships [21]. The NAP is one of the vertical programmes under the Disease Control Division, Department of Public Health, Ministry of Health and Sports. Myanmar has 124 ART centres and 173 decentralized sites for ART services.

Pyin Oo Lwin district is one of the seven districts in the Mandalay region. It has a combination of plain and hilly terrain. It has 5 townships and a population of 255,000. Under NAP, it has two ART centres (one at Pyin Oo Lwin and another at Mogoke) and one decentralized site. The ART centre at Pyin Oo Lwin is located at a district-level referral hospital.

#### Care of PLHIV under NAP (2014–2016)

**Figure 2.**
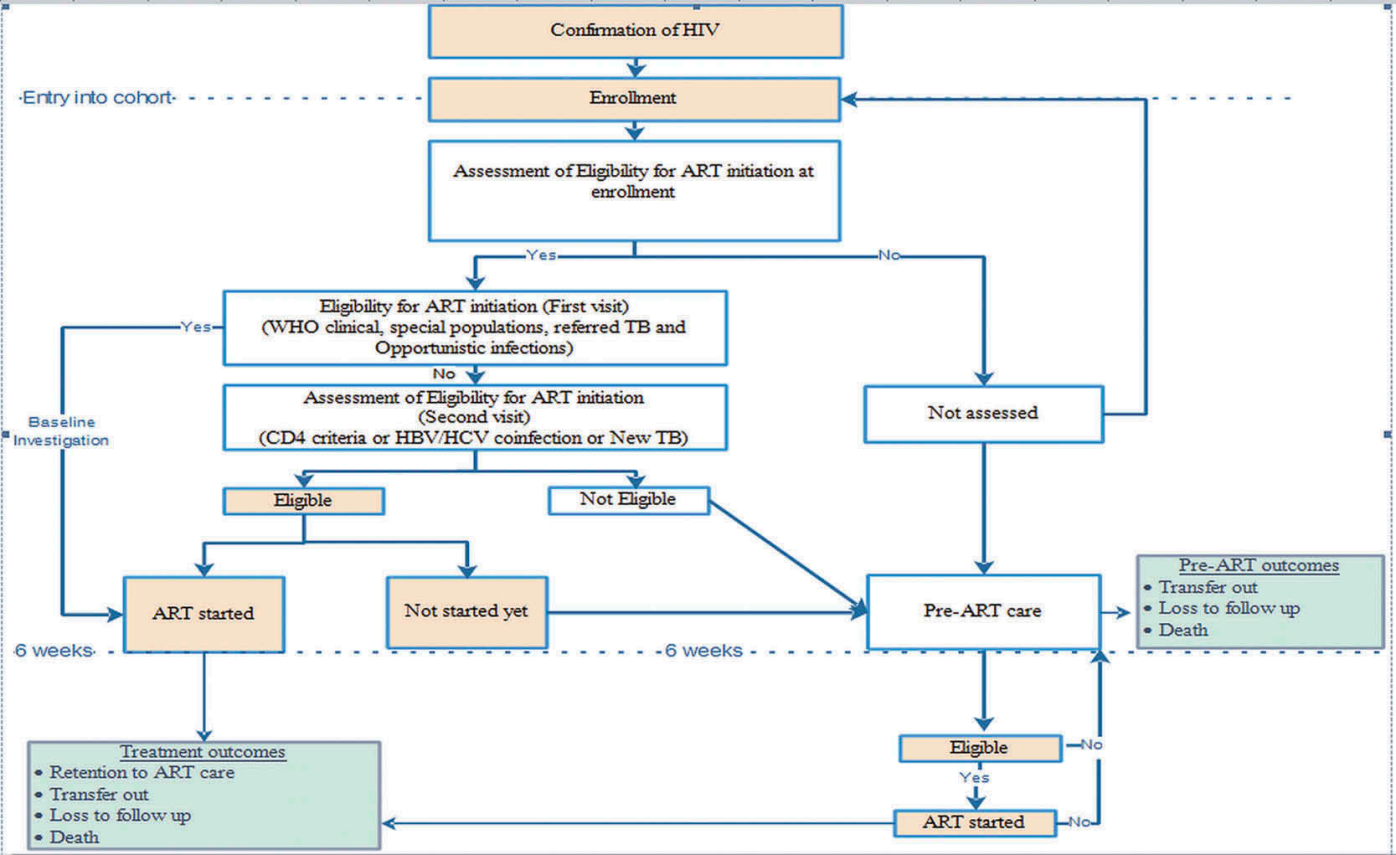
Flow chart on process of ART care for people living with HIV in ART clinics of the National AIDS Programme, Myanmar (2014–2016)*^†^. HIV = human immunodeficiency virus, ART = anti-retroviral therapy; AIDS = acquired immunodeficiency syndrome; HBV = hepatitis B virus; HCV = hepatitis C virus; TB = tuberculosis; WHO = World Health Organization *Box in pink colour is relevant during ‘test and treat’ strategy (implemented since September 2017). Box in white colour will not be relevant after introduction of ‘test and treat’ strategy.**^†^**Enrolled during 2014–2016 and followed up for ART initiation up to 5 December 2017; followed up until 31 March 2018 for attrition.

A flow chart describing the care of PLHIV is shown in Figure 2. A screening test for HIV, a confirmation test (‘three test’ policy) and enrolment was done at a decentralized site. Eligibility assessment and treatment initiation were done at an ART centre. Decentralized sites initiated ART only in key populations and TB-HIV co-infected patients. After enrolment, PLHIV were assessed for eligibility for ART, which was the presence of any one of the following: (i) WHO clinical staging 3 or 4; (ii) CD4 count below the threshold (≤ 350 cell/mm^3^ before July 2014, ≤ 500 cell/mm^3^ from July 2014); (iii) special populations (TB or Hepatitis B co-infection, sero-discordant couples, key populations, HIV-positive pregnant women).

PLHIV not assessed for eligibility, not eligible for ART and not started on ART despite eligibility within six weeks of enrolment were considered as ‘PLHIV under pre-ART care’. The PLHIV who were assessed and not yet eligible for ART were usually clinically healthy and followed up at three-monthly intervals. They received multivitamins, drugs for prophylaxis of opportunistic infections, and counselling for psychosocial support. Pre-ART outcomes (ART initiation, death, loss to follow-up, transfer out, still in pre-ART care) were recorded in the pre-ART register (black book).

If a patient was eligible for ART (at enrolment or during pre-ART care), baseline investigations along with counselling sessions (three times over two weeks) were prescribed and a follow-up date was given after two weeks. During this two-week period, cotrimoxazole preventive therapy (CPT) was started (if eligible: CD4 count < 350 per mm^3^ and/or WHO stage 3 or 4). Since the most common initial side effect of cotrimoxazole and anti-retroviral therapy is a rash, it was recommended to start CPT first and to initiate ART two weeks later if the individual does not develop a rash with cotrimoxazole. In cases of TB/HIV co-infection, if the patient was not on anti-TB treatment, anti-TB treatment was initiated first, followed by ART within two to eight weeks of starting anti-TB treatment. For those initiated on ART, information regarding CD4 count and WHO staging at the time of medical eligibility and date of medical eligibility were recorded in a pre-ART register (black book), ART register (red book) and ART card (white card). Patient visited the ART centre or the decentralized site on a monthly basis. Information regarding ART care was recorded in an ART register (red book) and ART card (white card). Though patients were screened for TB at enrolment and follow-up visits (sputum examination and chest radiograph if positive on symptom screen), isoniazid preventive therapy was not implemented during the study period. All the services were provided free of cost [22].

### Data variables, sources of data and data collection

The PLHIV were followed up to 5 December 2017 for ART initiation and up to 31 March 2018 (date of censoring) for attrition during ART.

The following variables were collected at enrolment from the pre-ART register: date of enrolment, date of HIV confirmation test, age, sex, risk factors for HIV infection, entry point to ART centre, literacy status, employment status, CPT/anti-TB treatment date. The time period until six weeks after the date of enrolment was taken as the enrolment period (‘at enrolment’). If initiated on ART, the following variables were additionally collected from the pre-ART register, ART register and white card: date of ART, date of medical eligibility, CD4 count at eligibility and WHO staging at eligibility.

Treatment outcomes (alive and still on treatment, transfer out, death and loss to follow-up) along with the dates of outcome were collected. Attrition was defined as death and loss to follow-up combined. The date of censoring was the date of outcome for PLHIV still on treatment. For death and loss to follow-up, more than 90 days after the last visit was taken as the date of outcome. For transfer out, the last visit date was considered.

### Data management and statistics

Data were single-entered into Excel (Microsoft, Redmond, WA, USA) and analysed using STATA (version 12.1 STATA Corp., College Station, TX, USA).

#### Derived variables

Year of enrolment was derived from the date of enrolment. PLHIV initiated on ART were classified as initiated within six weeks of enrolment or after six weeks of enrolment. Based on the date of enrolment and date of initiation of TB treatment, we classified TB as prevalent (on TB treatment at enrolment or initiated within six weeks of enrolment) or incident (initiated TB treatment after six weeks of enrolment) among the enrolled cohort. Similarly, CPT status was determined at enrolment.

Enrolment delay was calculated from the date of HIV confirmation to date of enrolment. Delay in eligibility and/or time period in pre-ART care was calculated among the ART cohort (date of medical eligibility was recorded only for those initiated on ART) from the date of enrolment to date of eligibility. In addition, ART initiation delay (from date of eligibility to date of ART initiation) and time to ART (from date of diagnosis to date of ART initiation) were also calculated among the ART cohort (Figure 1).

#### Data analysis

Baseline characteristics of the enrolled and ART cohort were summarized using mean (standard deviation – SD), median (inter-quartile range – IQR), frequency and proportions. Among the enrolled, ART initiation was summarized using frequency and proportions. Delay in days was summarized using median (IQR).

Risk factors associated with various types of delays (median was used as cut-off) were assessed using modified Poisson regression with robust variance estimates (forward stepwise method). Unadjusted and adjusted prevalence ratios (aPR) with 0.95 confidence interval (CI) were used to summarize (infer) the association.

Cumulative rates (%) and incidence density (per 100 person-years/per year [%]) of attrition (among ART cohort) were calculated. Survival analysis was undertaken to analyse the hazards of attrition after ART initiation. The risk time of PLHIV in ART care started from their date of ART initiation and ended with the date of attrition or censoring, whichever occurred earlier. Cox regression model was used to determine the association between various delays (one model for each delay) and attrition in ART care after adjusting for confounders. Proportional hazards assumption was confirmed by using ‘estat phtest’ function in STATA. Unadjusted and adjusted hazard ratios (0.95 CI) were used to summarize (infer) this association.

## Results

### Background and clinical characteristics

A total of 543 PLHIV were enrolled. Table 1 shows their background and clinical characteristics. Of 543, 319 (59%) were male, 194 (36%) belonged to the age group 25–34 years and 318 (59%) were heterosexuals. At enrolment, TB was prevalent in 52 (10%) people and 289 (53%) were on CPT. Among 52 with prevalent TB, 27 were already on anti-TB treatment at the date of enrolment and 25 were started on TB treatment within 6 weeks of the date of enrolment. Of 491 without prevalent TB, 6 (1.2%) developed incident TB post-enrolment.

**Table 1.** Socio-demographic and clinical characteristics of people living with HIV enrolled at a public sector ART centre in Pyin Oo Lwin, Myanmar (2014–2016) (N = 543).

Variables	N	(%)
**Total**	**543**	**(100.0)**
Sex		
Male	319	(58.7)
Female	224	(41.3)
Age in years		
18–24	48	(8.8)
25–34	194	(35.7)
35–44	191	(35.2)
≥ 45	110	(20.3)
** Mean (SD)**	36.7	(9.7)
Risk factors		
Heterosexual	318	(58.6)
MSM	3	(0.6)
FSW	7	(1.3)
IDU	61	(11.2)
Blood transfusion	12	(2.2)
Mother to child	1	(0.2)
Missing	141	(26.0)
Literate		
No	76	(14.0)
Yes	377	(69.4)
Missing	90	(16.6)
Employed		
No	125	(23.0)
Yes	331	(61.0)
Missing	87	(16.0)
Entry point		
VCT	180	(33.1)
STI	1	(0.2)
TB clinic	2	(0.4)
Outpatient	158	(29.1)
Inpatient	67	(12.3)
Private	24	(4.4)
Self-referred	18	(3.3)
Drug Treatment Unit	3	(0.6)
Missing	90	(16.6)
CPT at enrolment*		
No	254	(46.8)
Yes	289	(53.2)
TB at enrolment*		
No	491	(90.4)
Yes	52	(9.6)
Year of enrolment		
2014	170	(31.3)
2015	171	(31.5)
2016	202	(37.2)

HIV = human immunodeficiency virus, ART = anti-retroviral therapy, MSM = male who has sex with men, FSW = female sex worker, IDU = intravenous drug users, STI = sexually transmitted infection, TB = tuberculosis, VCT = voluntary counselling and testing, CPT = cotrimoxazole preventive therapy

*Until six weeks from date of enrolment.

Out of 543, 373 (69%) were found to be eligible and initiated on ART and 245 (45%) were initiated at enrolment. Baseline characteristics of the 373 PLHIV initiated on ART are given in Table 2. Of 373, 78 (21%) had WHO clinical stage of more than 2 and the median CD4 count at was 206 (IQR: 109, 332) per mm^3^.

**Table 2.** Socio-demographic and clinical characteristics of people living with HIV initiated on ART at a public sector ART centre in Pyin Oo Lwin, Myanmar (2014–2016) (N = 373)^†^.

Variables	n	(%)
**Total**	**373**	**(100.0)**
Sex		
Male	212	(56.8)
Female	161	(43.2)
Age in years		
18–24	28	(7.5)
25–34	122	(32.7)
35–44	137	(36.7)
≥ 45	86	(23.1)
**Mean (SD)**	**37.6**	**(9.7)**
Risk factors		
Heterosexual	269	(72.1)
MSM	2	(0.5)
FSW	4	(1.1)
IDU	48	(12.9)
Blood transfusion	10	(2.7)
Mother to child	1	(0.3)
Missing	39	(10.5)
Literate		
No	62	(16.6)
Yes	309	(82.8)
Missing	2	(0.6)
Employed		
No	103	(27.6)
Yes	268	(71.8)
Missing	2	(0.6)
Entry point		
VCT	125	(33.5)
STI	1	(0.3)
TB clinic	2	(0.5)
Outpatient	145	(38.9)
Inpatient	58	(15.6)
Private	22	(5.9)
Self-referred	18	(4.8)
Drug Treatment Unit	1	(0.3)
Missing	1	(0.3)
WHO stage		
Stage 1	155	(41.6)
Stage 2	75	(20.1)
Stage 3	61	(16.4)
Stage 4	17	(4.6)
Missing	65	(17.4)
CD4 count		
<50	24	(6.4)
50–99	53	(14.2)
100–199	99	(26.5)
200–349	104	(27.9)
350–499	48	(12.9)
≥ 500	35	(9.4)
Missing	10	(2.7)
**Median (IQR)**	**206**	**(109, 332)**
Year of enrolment		
2014	93	(24.9)
2015	127	(34.1)
2016	153	(41.0)

HIV = human immunodeficiency virus, ART = anti-retroviral therapy, MSM = male who has sex with men, FSW = female sex worker, IDU = intravenous drug users, STI = sexually transmitted infection, TB = tuberculosis, VCT = voluntary counselling and testing, WHO = World Health Organization; CPT = cotrimoxazole preventive therapy

**^†^**Enrolled during 2014–2016 and followed up for ART initiation up to 5 December 2017.

*Until six weeks from date of enrolment.

### Time to ART: magnitude and risk factors

Within 7 days of diagnosis, 26 (8%) PLHIV were initiated on ART. The median enrolment delay and ART initiation delay were 4 days (IQR: 1 to 14 days) and 20 days (IQR: 13 to 36 days), respectively. The median time to ART was 41 days (IQR: 25 to 92 days). Assuming ‘test and treat’ during the study period, we calculated the median time to ART after excluding the delay in eligibility and/or time interval in pre-ART care which was 29 days (IQR: 18 to 55 days) (Table 3). This was nothing but enrolment delay and ART initiation delay combined.

**Table 3.** Time taken (days) for ART initiation from HIV diagnosis among people living with HIV enrolled at a public sector ART centre in Pyin Oo Lwin, Myanmar (2014–2016).

Time taken (days) in care cascade from HIV diagnosis	Eligible and assessed*	Median (IQR)	Range
Enrolment delay (a)	472	4 (1, 14)	0, 1105
Eligibility delay and/or time interval in pre-ART care (b)	373	4.5 (0, 13.3)	0, 559
ART initiation delay (c)	373	20 (13, 36)	0, 673
Time to ART (a + b + c)	330	39 (24, 74)	0, 1105
Time to ART after excluding the delay in eligibility and/or time interval in pre-ART care (a + c)	330	29 (18, 55)	0, 1105

HIV = human immunodeficiency virus, ART = anti-retroviral therapy; IQR = inter-quartile range

*543 were enrolled and 373 were initiated on ART as on 5 December 2017. Those who were eligible and dates were available were included.

No baseline factors were significantly associated with enrolment delay and ART initiation delay (data not shown). Prevalent TB at enrolment and CD4 count at ART eligibility were significantly associated with a long time to ART (≥ 30 days, after excluding the delay in eligibility and/or time interval in pre-ART care). When compared to PLHIV without prevalent TB, those with prevalent TB had a 42% higher chance of delay (aPR [0.95 CI]: 1.42 [1.13, 1.79]). When compared to PLHIV with a CD4 count less than 50, those with a count ≥ 500 had a 59% higher chance of delay (aPR [0.95 CI]: 1.59 [1.00, 2.52]). Other factors like year and literacy were not significantly associated with delay (Table 4).

**Table 4.** Factors associated with long time to ART* (≥ 30 days) after excluding the time interval in pre-ART care among people living with HIV initiated on ART at a public sector ART centre in Pyin Oo Lwin, Myanmar (2014–2016) (N = 373)^†^.

Variables		Crude PR (0.95 CI)	Adjusted PR^#^ (0.95 CI)
Entry point at enrolment			
	VCT	Ref	Ref
	STI/TB/Drug Treatment Unit	-^	-^
	Outpatient	0.87 (0.71, 1.08)	0.86 (0.70, 1.06)
	Inpatient	0.94 (0.72, 1.22)	0.89 (0.69, 1.16)
	Private/self-referred	0.70 (0.47, 1.03)	0.70 (0.48, 1.03)
	Missing	-^	-^
Prevalent TB at enrolment			
	No	Ref	Ref
	Yes	1.39 (1.12, 1.71)**	1.42 (1.13, 1.79)**
CD4 count (microlitre) at ART start			
	<50	Ref	Ref
	50–99	1.06 (0.66, 1.70)	1.22 (0.77, 1.93)
	100–199	1.11 (0.72, 1.72)	1.25 (0.83, 1.91)
	200–349	1.15 (0.75, 1.78)	1.34 (0.87–2.05)
	350–499	1.13 (0.70, 1.80)	1.36 (0.85, 2.19)
	≥ 500	1.31 (0.82, 2.10)	1.59 (1.00, 2.52)**
	Missing	-^^^	-^^^
Constant		-	0.46 (0.30, 0.70)

HIV = human immunodeficiency virus, ART = anti-retroviral therapy, STI = sexually transmitted infection, TB = tuberculosis, VCT = voluntary counselling and testing, PR = prevalence ratio, aPR = adjusted prevalence ratio, CI = confidence interval

**^†^**Enrolled during 2014–2016 and followed up for ART initiation up to 5 December 2017.

*Combination of enrolment delay and ART initiation delay. We categorized the delay based on the median value.

**^#^**Modified Poisson regression with robust variance estimates (forward stepwise method); other variables at enrolment (age, sex, HIV risk factor, literacy, employment status, cotrimoxazole preventive therapy status, year of enrolment) and at ART eligibility (WHO clinical staging) were considered in the model. However, they were excluded by the model as they did not significantly improve the model prediction.

^^^Limited sample (n ≤ 10) in this sub-category.

**p < 0.05

### Attrition while on ART: magnitude and risk factors

**Figure 3.**
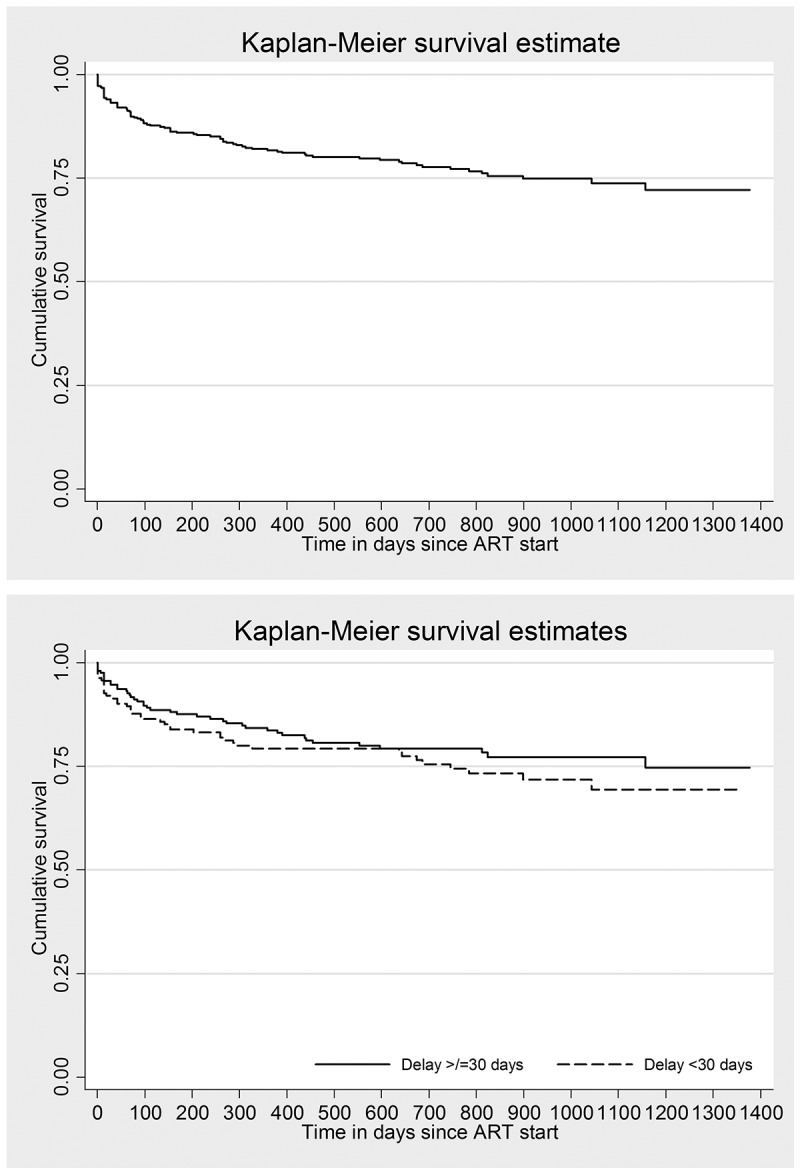
Kaplan Meier survival curve^^^ among people living with HIV initiated on ART* at a public sector ART centre in Pyin Oo Lwin, Myanmar (2014–2016): overall and stratified by time to ART after excluding the delay in eligibility and/or time interval in pre-ART care*^†^. HIV = human immunodeficiency virus, ART = anti-retroviral therapyLog rank test P value = 0.325, ^^^Event of interest is attrition which includes loss to follow-up and death.*Combination of enrolment delay and ART initiation delay, categorized based on the median value.^†^Enrolled during 2014–2016 and followed up for ART initiation up to 5 December 2017; followed up until 31 March 2018 for attrition.

The outcomes of PLHIV stratified by ART initiation within six weeks of enrolment and after six weeks of enrolment are provided in Table 5. Among 373, cumulative attrition was seen in 85 (23%) which was contributed by 41 (11%) deaths and 44 (11.8%) loss to follow-up. The annual incidence density of attrition was 12.8% (0.95 CI: 10.2, 15.7). Attrition was common in first 100 days of ART. (Table 6 and Figure 3)

**Table 5.** Outcomes among people living with HIV initiated on ART at a public sector ART centre in Pyin Oo Lwin, Myanmar (2014–2016) (N = 373)^†^.

Outcomes	Initiated at enrolment*		Initiated after enrolment		Overall	
	n	(%)	n	(%)	n	(%)
Total	245	(100)	128	(100)	373	(100)
Still on treatment	151	(61.6)	88	(66.7)	239	(64.1)
Transferred out	36	(14.7)	13	(10.2)	49	(13.1)
Death**	27	(11.0)	14	(10.9)	41	(11.0)
Loss to follow-up**	31	(12.7)	13	(10.2)	44	(11.8)

HIV = human immunodeficiency virus, ART = anti-retroviral therapy

**^†^**Enrolled during 2014–2016 and followed up for ART initiation up to 5 December 2017; followed up until 31 March 2018 for attrition.

*Until six weeks from date of enrolment.

**Included under attrition.

**Table 6.** Cumulative incidence of attrition rate among people living with HIV initiated on ART at a public sector ART centre in Pyin Oo Lwin, Myanmar (2014–2016) (N = 373)^†^.

Cohort	Person-days of follow-up	Attrition^^^	Incidence density per year (%)	(0.95 CI)
Total	237,628	83	12.8	(10.2, 15.7)
0–100 days	33,545	43	46.7	(34.7, 63.1)
101–200 days	30,011	8	9.9	(4.7, 19.3)
> 200 days	174,072	32	6.6	(4.8, 9.5)

HIV = human immunodeficiency virus, CI = confidence interval

**^†^**Enrolled during 2014–2016 and followed up for ART initiation up to 5 December 2017; followed up until 31 March 2018 for attrition.

^^^Includes loss to follow-up and death.

After adjusting for potential confounders (CD4 count, WHO staging, literacy and employment status, entry point, HIV risk factors, year, prevalent TB, CPT use at enrolment, age and sex), the association of various delays before ART and attrition have been summarized in Table 7. The time to ART (after excluding the delay in eligibility and/or time interval in pre-ART care) was not significantly associated with attrition. However, long enrolment delay (≥ 5 days) was significantly associated with less attrition.

**Table 7.** Confounder adjusted association between various delays before ART initiation and attrition (death and loss to follow-up) among people living with HIV initiated on ART at public sector ART centre in Pyin Oo Lwin, Myanmar (2014–2016) (N = 373)^†^.

Delay type*	Attrition**	HR (0.95 CI)	aHR (0.95 CI)^#^
Enrolment delay (a)			
Yes	7.4	0.64 (0.41, 0.99)	0.60 (0.37, 0.99)^^^
No	12.1	Ref	Ref
Eligibility delay and time on pre-ART care (b)			
Yes	12.4	0.93 (0.60, 1.43)	0.72 (0.42, 1.24)
No	13.1	Ref	Ref
ART initiation delay (c)			
Yes	12.0	0.88 (0.57, 1.35)	0.71 (0.42, 1.20)
No	13.5	Ref	Ref
Time to ART (a + b + c)			
Yes	10.6	0.72 (0.47, 1.12)	0.72 (0.44, 1.17)
No	15.3	Ref	Ref
Time to ART after excluding the delay in eligibility and/or time interval in pre-ART care (a + c)			
Yes	11.7	0.81 (0.52, 1.24)	0.67 (0.40, 1.13)
No	14.2	Ref	Ref

HIV = human immunodeficiency virus, HR = hazard ratio; aHR = adjusted hazard ratio; CI = confidence interval

**^†^**Enrolled during 2014–2016 and followed up for ART initiation up to 5 December 2017; followed up until 31 March 2018 for attrition.

*Median delay was used to categorize each delay type.

**Incidence density per year (%)/incidence density per 100 person-years, attrition includes death and loss to follow-up.

**^#^**Cox regression (enter method); adjusted for CD4 count, WHO staging, literacy and employment status, entry point, HIV risk factors, year, prevalent TB, CPT use at enrolment, age and sex.

^^^p < 0.05

## Discussion

This is the first study from Myanmar looking at time to ART from HIV diagnosis. The major strength of this study is that we documented the entire HIV care cascade from enrolment to treatment outcome and attempted to study time to ART in a hypothetical ‘test and treat’ scenario. We also made an attempt to assess the association between pre-ART delays and ART treatment outcomes. There were some key findings.

First, two-thirds of enrolled PLHIV were eligible and initiated on ART during the follow-up period and half were initiated on ART within six weeks of enrolment. It was related to a policy of assessment for eligibility for ART initiation during the study period. Though we do not know the status of those not on ART, a previous study in Myanmar has detected alarmingly high attrition during pre-ART care [15]. There is evidence that PLHIV often encountered barriers in accessing ART-related services (both before and after ART uptake) even in contexts where these services are freely available [6,23]. The ‘Test and treat’ strategy that has been implemented in Myanmar eliminates the need for pre-ART care [18]; however, the barriers to accessing free ART was beyond the scope of this study.

Second, the time to ART (after excluding the delay in eligibility and/or time interval in pre-ART care) was around a month and was largely contributed by ART initiation delay. Less than 1 in 10 were initiated on ART within a week of diagnosis. The time to ART was lower than reported elsewhere globally and similar to a study from Malawi (for the year 2011) [5–11]. However, this has to be interpreted with caution as the eligibility criteria might vary based on the study site and period.

ART initiation delay was probably related to the waiting time for completing two weeks of CPT (CPT at enrolment was 53%) and two to eight weeks of anti-TB treatment (10% had prevalent TB at enrolment). Prevalent TB at enrolment was also an independent predictor of long time to ART (after excluding the delay in eligibility and/or time interval in pre-ART care). With ‘test and treat’, PLHIV with a higher CD4 count at enrolment will become eligible for ART, reducing the number of people on CPT and with prevalent TB at enrolment significantly. This will help reduce the ART initiation delay. However, all PLHIV after eligibility also received counselling sessions for two weeks before ART initiation [22]. Under ‘test and treat’, WHO recommends ART as soon as possible if the patient provides consent, and this issue of two weeks’ counselling needs to be addressed by the programme [17].

Third, a high CD4 count has been previously associated with delay in enrolment and long time to ART [7,9]. The long time to ART may be explained by delay in becoming eligible for ART. In our study, a high CD4 count was associated with long time to ART after excluding the delay in eligibility and/or time interval in pre-ART care. With ‘test and treat’, many PLHIV with a high CD4 count will be eligible for ART. This requires attention by the programme.

Fourth, the annual attrition rate was higher than that reported previously in a large cohort of PLHIV managed by NAP in collaboration with a non-government organization (12.8% versus 8.8%). High attrition in the initial 100 days was similar to this previous study where attrition was significantly higher in the initial 180 days [14]. Time to ART (after excluding the delay in eligibility and/or time interval in pre-ART care) was not associated with attrition. Enrolment delay (≥ 5 days) was a protective factor for attrition. This should be interpreted with caution. This analysis was limited by the exclusion of factors like socio-economic status, marital status, sero-discordant status, alcohol/substance abuse, ART regimen, distance of residence from ART centre, baseline weight and patient’s knowledge and attitude from the adjusted analysis. These were not collected.

Finally, in addition to not collecting an exhaustive list of confounders, there were other limitations as well. We were not able to describe the pre-ART care cascade (how many were assessed at enrolment; of those assessed, how many were eligible; and of those eligible, how many were initiated on ART) including the pre-ART outcomes. Details regarding eligibility (staging and CD4 count) were recorded only if a PLHIV was initiated on ART. Therefore, we did not have information on CD4 counts and WHO staging at enrolment. The programme needs to improve recording (especially CD4 counts and WHO staging at enrolment) as it has moved on to the ‘test and treat’ strategy.

### Future research

Fox MP et al. in their systematic review (studies from 2008 to February 2015) found that the evidence on interventions in sub-Saharan Africa to improve rate and time of ART uptake was limited and of mixed quality. They recommended more implementation research to improve uptake in a manner that is efficient for providers and effective for PLHIV without affecting treatment outcomes [24]. The findings of this study provide the evidence base for more implementation research to reduce time to ART in a ‘test and treat’ setting.

This study was limited to one ART centre in the Mandalay region; one of the better-performing centres. Similar studies are urgently recommended in other part of Myanmar before a policy decision can be made. Qualitative studies are recommended in Myanmar to explore the provider and patient perspectives into barriers to early ART uptake.

## Conclusion

We studied the time to ART among PLHIV in a three-year period before the ‘test and treat’ approach was implemented in Myanmar. This provides necessary baseline information to support the national programme to reduce time to ART during the implementation of ‘test and treat’. The NAP appears to be on track to implement ‘test and treat’ subject to interventions to reduce the ART initiation delay by addressing the issue of ART counselling which itself induces a two-to-three-week delay and addressing the high-risk group of ‘people with high CD4 counts’. We recommend including routine indicators within the programme to measure linkage to care (enrolment) among all diagnosed, ART uptake among those enrolled (both proportion and time) and early ART outcomes (in first 100 days) as per the recently recommended updates in the HIV care cascade [19].

## Data Availability

The dataset used in this study (STATA data file) along with the programme file used for analysis (STATA do file) and codebook have been provided as a supplementary annex **(S Annex)**.

## Paper context

In Myanmar, time to anti-retroviral therapy from HIV diagnosis has not been studied. In the anti-retroviral therapy centre of Pyin Oo Lwin, the median time to anti-retroviral therapy after excluding the period in pre-anti-retroviral therapy care was 29 days and largely contributed by delay in ART initiation after eligibility. As Myanmar has begun implementing ‘test and treat’, this calls for interventions to reduce delay in starting anti-retroviral therapy after enrolment.
