# Supplementary material for: Time to anti-retroviral therapy among people living with HIV enrolled into care in Myanmar: how prepared are we for ‘test and treat’?
Source: Glob Health Action. 2018 Nov 30;11(1):1520473. doi: 10.1080/16549716.2018.1520473 (PMC6282424; doi:10.1080/16549716.2018.1520473)
Supplement: Supplemental Material [file ZGHA_A_1520473_SM3028.zip › codebook.doc]

Title: **Cascade of care among PLHIV enrolled at ART centers under National AIDS programme in Pyin Oo Lwin District, Myanmar (2014-2016)**

PI:KYAW ZIN LINN (Data entry code book)

| **Field name** | **Field label** | **Field type** | **Field length** | **Field value** | **Value labels** | **Comment** |
| --- | --- | --- | --- | --- | --- | --- |
| code | Code identifier | Text | 6 |  |  | Key Unique. No enter field. manual entering |
| year | Year of enrollment | I | 2 |  |  |  |
| Dd | date of HIV diagnosis  confirmation | Date | 10 | 01/06/2011-31/12/2016  09/09/2009 |  | Range of legal date of HIV diagnosis confirmation  For missing date |
| enrollda | Enrolment date | Date | 10 | 01/01/2014-31/12/2016 |  | Range of legal date of enrollment |
| eli | Eligibility or Not Eligible | I | 1 | 1  2  9 | Eligible  Not eligible  Missing |  |
| elidate | If eligible, date of eligibility | Date | 10 | 01/01/2014-14/11/2017  09/09/2009  01/01/2000 | Missing  Not applicable |  |
| ARTstarted | ART started or ART started | I | 1 | 1  2  9  8 | ART started  Not ART started  Missing  Censored |  |
| stdate | If ART started after enrolment, date of ART initiation | Date | 10 | 01/01/2014-31/12/2017  1/1/2099  09/09/2009  01/01/2000 |  | Range of legal date of ART initiation  Censored date  Missing  Not applicable |
| Sex | Gender | I | 1 | 1  2 | Male  Female |  |
| age | Age (in completed years) | I | 2 | 13-98  99 |  | Enter 99 if age not recorded |
| agegrp | Group of age | I | 1 | 1  2  3  4 | 16-24  25-34  35-44  ≥45 |  |
| Riskfact |  | Risk factors | I | 1 | 1  2  3  4  5  7  9 | Heterosexual  MSM  FSW  IDU  Blood transfusion  Unknown  Missing |
| Illiterate | Literate | I | 1 | 1  2  9 | Yes  No  Missing |  |
| Employed | Employed | I | 1 | 1  2  9 | Yes  No  Missing |  |
| Entrypoi | Entry point | I | 2 | 1  3  4  5  6  7  9  10  99 | VCT  STI  TB clinic  Outpatient  Inpatient  Private  Self-referred  Drug Treatment unit  Missing |  |
| Whostagi | WHO staging | I | 1 | 1  2  3  4  9 | Stage 1  Stage 2  Stage 3  Stage 4  Missing |  |
| Cpt | Cotrimoxazole prophylaxis therapy | I | 1 | 1  2 | Yes  No |  |
| cptbaart | Cotrimoxazole Before and after enrollment and ART initiation | I | 1 | 1  2  3  8 | Before enrollment  Between enrollment and ART  After ART  Not applicable |  |
| Cd4atsta | CD4 count | I | 4 | 1-2000  9999 | Missing | Enter 9999 if CD4 count not recorded |
| Cd4gp | Category of CD4 count | I | 1 | 1  2  3  4  5  6  9 | <50  50-99  100-199  200-349  350-499  ≥500  Missing |  |
| Dateoftb | Date of TB di agnosis | Date | 10 | 01/01/2014-14/11/2017 |  |  |
| tb | TB presence or not | I | 1 | 1  2 | Yes  No |  |
| tbbaart | TB Before and after enrollment and ART initiation | I | 1 | 1  2  3  8 | Before enrollment  Between enrollment and ART  After ART  Not applicable |  |
| Lvdate | Last visit date to ART clinic among people on ART | Date | 10 | 01/01/2014-31/03/2018  09/09/2009  01/01/2000 |  | Range of last visit date to ART clinic  Missing  Not applicable |
| Fupdate | Next follow up visit date to ART clinic among people on ART | Date | 10 | 01/01/2014-31/08/2018  09/09/2009  01/01/2000 |  | Range of next visit date to ART clinic  Missing  Not applicable |
| ioc | ART outcome | I | 1 | 1  2  3  4  8  9 | Still on treatment  Death  Loss to follow up  Transfer out  Not applicable  Missing |  |
| ttde | Time taken between date of diagnosis and date of enrollment | I  (days) | 4 | in days |  | Compute ttde=enrollda-dd |
| ttee | Time taken between date of enrollment and date of eligible | I  (days) | 4 | in days |  | Compute ttde= eligibildate-enrollda |
| artdel | Time taken between date of eligible and date of Treatment initiation | I  (days) | 2 | in days |  | Compute artdel= stdate -eligibildate |
| Eli6wks | Eligible for ART within 6 weeks of enrollment  (Delay in Eligibility) | I | 1 | 1  2  8 | Yes  No | if ttee<=6 weeks then  Eli6wks=1  if ttee>6 weeks then  Eli6wks=2 |
| ARTattri | Attrition of ART patients | I | 1 | 1  2 | Yes  No | recode oc=2 or oc=3 into attrition=1  recode oc=1 or oc=4 into attrition=1  9=missing |
